# Reduction of an Unusual Dislocation of Both Bones of the Fore Arm at the Elbow Joint, Complicated with the Fracture of the Internal Condyle of the Humerus and of the Tip of the Caronoid Process of the Ulna, Two Months after the Receipt of the Injury

**Published:** 1867-05

**Authors:** A. W. Griggs

**Affiliations:** West Point, Ga.


### ARTICLE II.

*Reduction of an unusual Dislocation of both Bones of the Fore Arm at the Elbow Joint, complicated with the fracture of the internal condyle of the humerus and of the tip of the Caronoid process of the Ulna, two months after the receipt of the injury.* Reported by A. W. Griggs, M. D., of West Point, Ga.

On the 6th day of December 1866, Dr. H. G. Tate, my co-partner in the practice, was called to see Mr. W. H. Clark, a highly respectable merchant of this city, and found him suffering from an injury received fifteen hours previously, by a fall from a vehicle. The accident occurred the evening before, on the Hamilton road, four miles distant from West Point. It appears that the animal working to the vehicle, rushed precipitately forward, and the vehicle came in contact with a stump,—thus throwing the patient from his seat to the ground. He was considerably stunned; and whisky was obtained, and very freely administered by his companions. As soon as reaction began, he made great complaint of his left arm. They continued the whisky, which was all that they had in the way of medicine, and brought him to the east side of the river, to the house of a friend in that part of the city, where he remained during the night, without medical attention. Dr. Tate found the left arm greatly tumefied: the soft tissues so much strutted that it was impossible to make out a correct diagnosis. He ordered cold water to be constantly applied, and placed the patient under the strictest hygienic regimen—(of course antiphlogistic). On the 8th, the 3d day after the injury was received, I visited the case with Dr. Tate, and found the arm enormously swollen, and considerably ecchymosed. I had fears that gangrene would take place; but perseverence in the plan already inaugurated caused a subsidence of the aggravated symptoms in the course of twelve or fifteen days, and we could begin to get an insight into the nature of the case. It was now our opinion, that there was a lateral displacement of the ulna inwards, with a slight fracture of the coronoid process,—thus admitting of the projection of the articulating surface of humorus upon the anterior and inner aspect of the elbow joint. There was also evident fracture of the internal condyle of the humerus, and the head of the radius was thrown behind the eminentia capitata. The arm could not be flexed, and therefore was very painful and inconvenient to the patient. Mr. C. had lost so much time from his business, that he entered for *duty* before having any operation performed for his relief. We frequently advised him to do so; but the press of his avocation caused him to continue to procrastinate. Finally, being worn out with suffering and inconvenience occasioned by the condition of the injured member, he resolved to submit himself again to his physicians for remedy.

We advised him of the probable danger that was connected with every such procedure; and on the 5th day of February 1866, precisely two months from the time of the receipt of the injury, Dr. Tate and myself, with two other professional gentlemen of this city, undertook the reduction of the dislocation. The muscles and ligaments about the joint were very rigid and resisting; and the arm somewhat swollen, especially the wrist and hand, of which he had greatly complained all the time. Chloroform was administered by Dr. Gardner; and Dr. Tate placed his knee against the joint, and with the assistance of Dr. Oslin and myself, he began, gradually, efforts to flex the arm. This plan failed, notwithstanding that it was repeated by Dr. Oslin, and, also, by myself. Chloroform did not produce its usual symptoms, overcoming the tonic contractions of the muscles. We now tried another plan. Dr. Tate grasped the fore arm, and an assistant made traction at the end of a short bandage, one end of which was fastened above the wrist, while Dr. Oslin and I placed our fore arms at the joint—the one above, and the other below,—thus making a double fulcrum. We were so situated as to use great force; and the dislocation was, in this wise, reduced. The arm supporting the inferior extremity of the humerus, produced counter extension; and the arm acting upon the superior extremities of the bones of the fore arm, assisted by separating their new detachment; also, disengaged them from their entanglement against the irregular surface with which they were in contact. Gradual and continued extension was made in the way above described, by Dr. Tate and his associates, pulling the arm first in the axis in which it was found, and then bringing it to a right angle. This being done, an angular splint was applied; and for the better security from accident, strips of adhesive plaster were passed from the waist to the shoulder of the opposite side, an anodyne administered, and cold water ordered to be constantly applied. The symptoms which occurred subsequently to the reduction, were by no means so iminent as those which appeared after the receipt of the primary injury. The patient still carries his arm in a support, but can use it a little; he can nearly straighten it; and can also carry the hand to the mouth by the assistance of his other; he has good use of his fingers; and we think that, notwithstanding the many difficulties connected with the case, the result will prove a success.

Mr. H. is low, heavy-set man, enjoys fine health, and is very athletic, and about 37 years of age. We hesitated as to the propriety of undertaking so serious a measure. In the first place, we might fracture the olecranon process, or break up unions just formed; secondly, we feared injury to important blood vessels, and also the renewal of active inflammation. We were, however encouraged to attempt the reduction from a successful experiment previously made by Dr. W. F. Westmoreland, the Prof. of Surgery in the Atlanta Medical College, which consisted in reducing a dislocation of both bones of the fore arm backward, five months after the accident.

